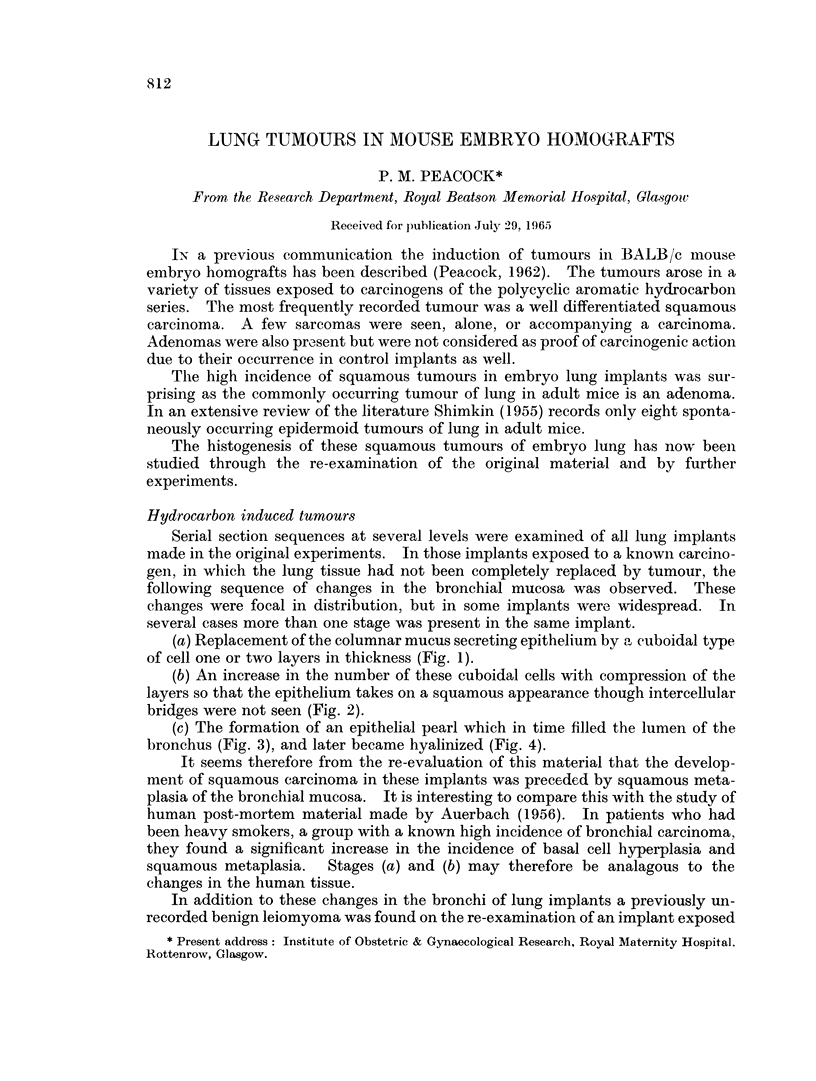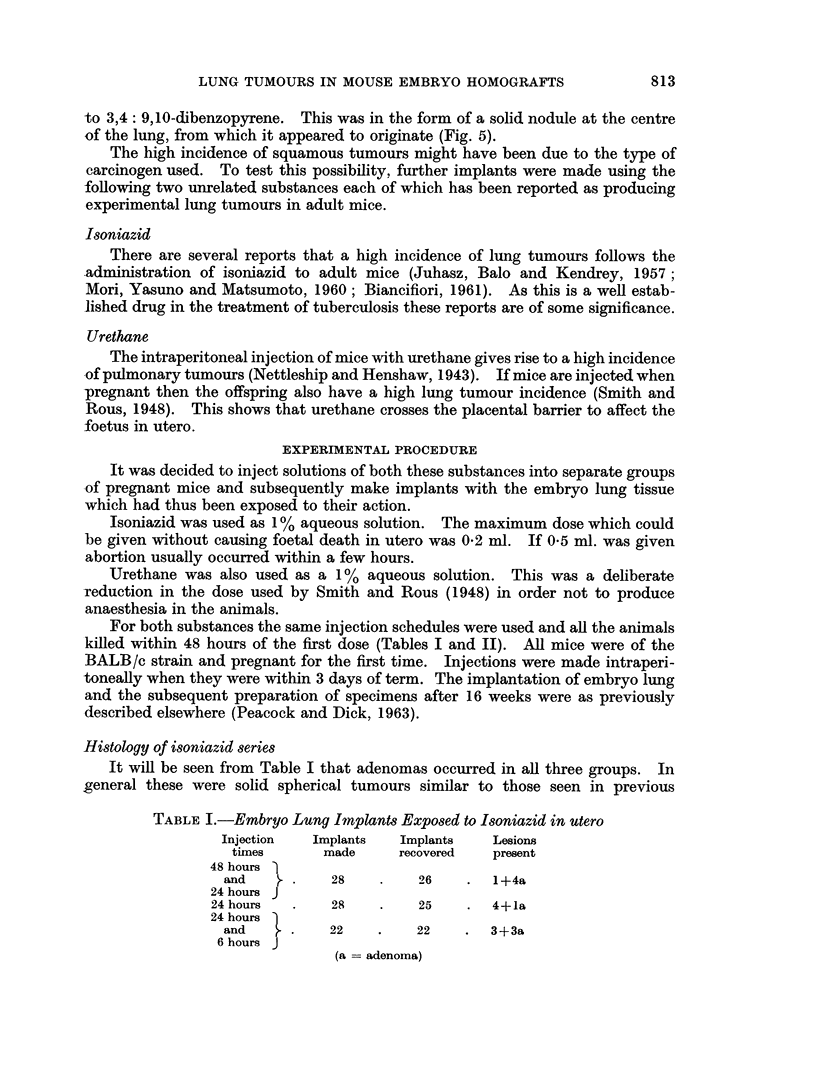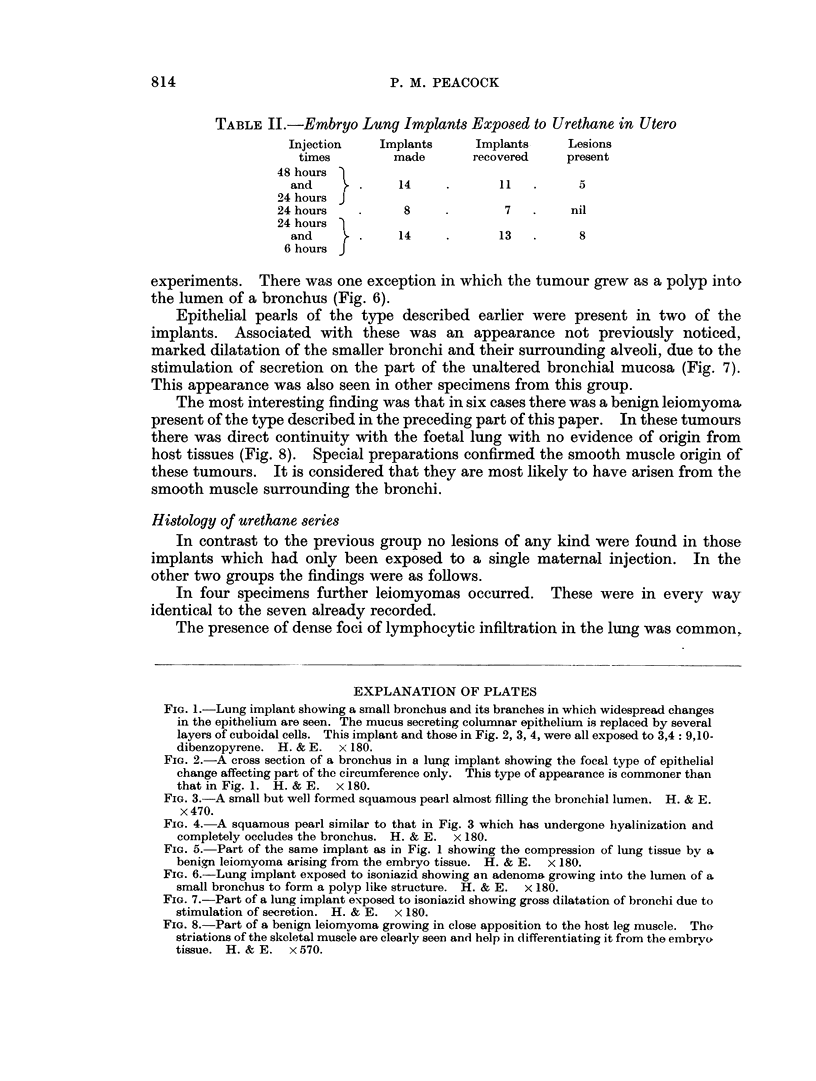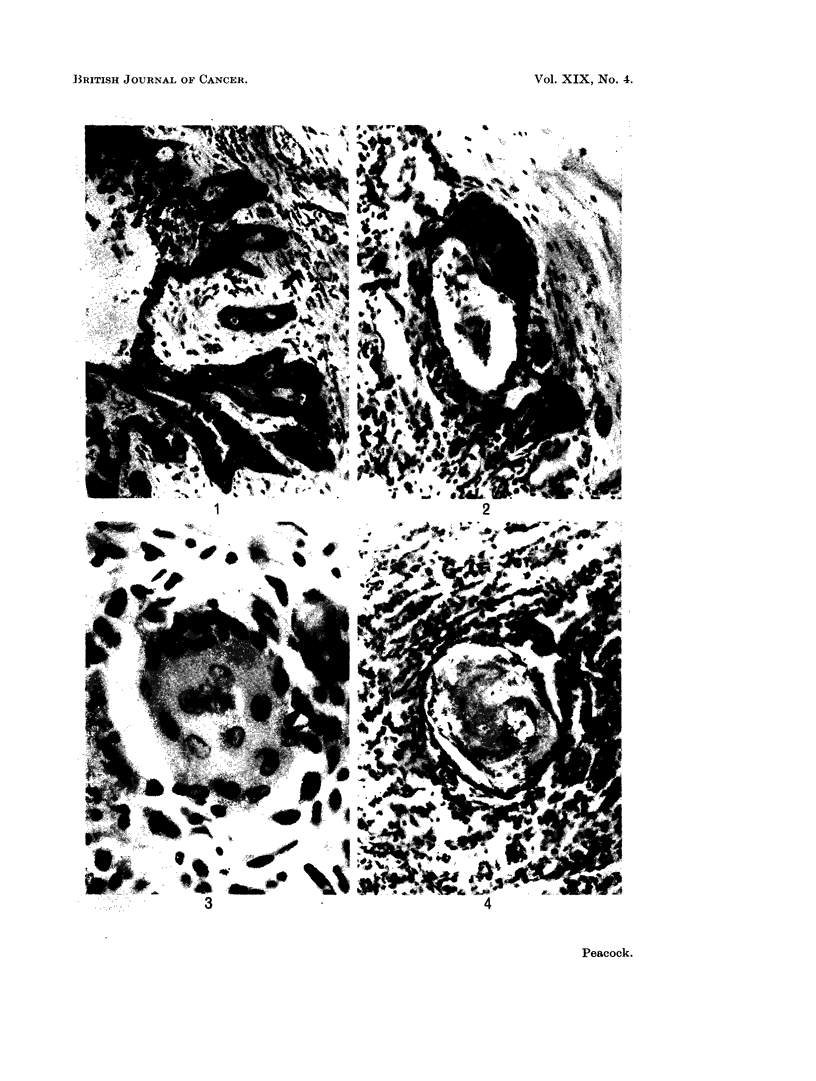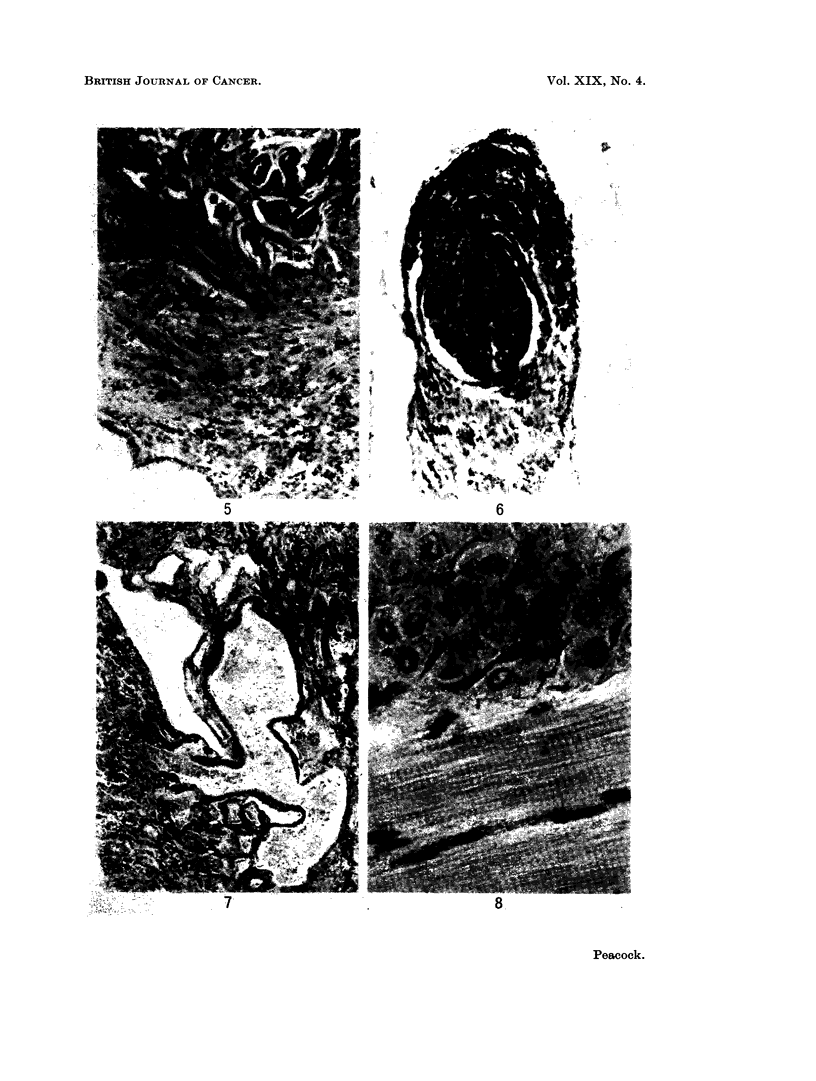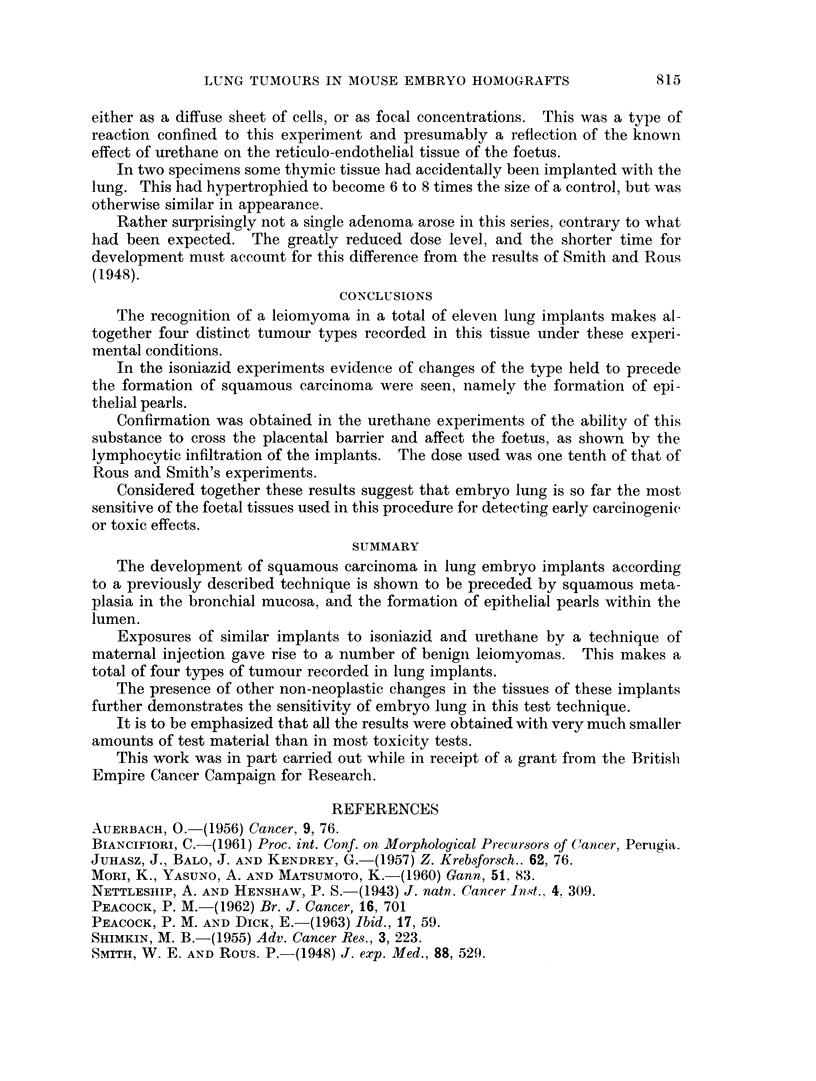# Lung tumours in mouse embryo homografts.

**DOI:** 10.1038/bjc.1965.93

**Published:** 1965-12

**Authors:** P. M. Peacock

## Abstract

**Images:**


### LUNG TUMOURS IN MOUSE EMBRYO HOMOGRAFTS

P. M. PEACOCK*

*From the Research Department, Royal Beatson Memorial Hospital, Glasgow*

Received for publication July 29, 1965

IN a previous communication the induction of tumours in BALB/c mouse embryo homografts has been described (Peacock, 1962). The tumours arose in a variety of tissues exposed to carcinogens of the polycyclic aromatic hydrocarbon series. The most frequently recorded tumour was a well differentiated squamous carcinoma. A few sarcomas were seen, alone, or accompanying a carcinoma. Adenomas were also present but were not considered as proof of carcinogenic action due to their occurrence in control implants as well.

The high incidence of squamous tumours in embryo lung implants was surprising as the commonly occurring tumour of lung in adult mice is an adenoma. In an extensive review of the literature Shimkin (1955) records only eight spontaneously occurring epidermoid tumours of lung in adult mice.

The histogenesis of these squamous tumours of embryo lung has now been studied through the re-examination of the original material and by further experiments.

*Hydrocarbon induced tumours*

Serial section sequences at several levels were examined of all lung implants made in the original experiments. In those implants exposed to a known carcinogen, in which the lung tissue had not been completely replaced by tumour, the following sequence of changes in the bronchial mucosa was observed. These changes were focal in distribution, but in some implants were widespread. In several cases more than one stage was present in the same implant.

(*a*) Replacement of the columnar mucus secreting epithelium by a cuboidal type of cell one or two layers in thickness (Fig. 1).

(*b*) An increase in the number of these cuboidal cells with compression of the layers so that the epithelium takes on a squamous appearance though intercellular bridges were not seen (Fig. 2).

(*c*) The formation of an epithelial pearl which in time filled the lumen of the bronchus (Fig. 3), and later became hyalinized (Fig. 4).

It seems therefore from the re-evaluation of this material that the development of squamous carcinoma in these implants was preceded by squamous metaplasia of the bronchial mucosa. It is interesting to compare this with the study of human post-mortem material made by Auerbach (1956). In patients who had been heavy smokers, a group with a known high incidence of bronchial carcinoma, they found a significant increase in the incidence of basal cell hyperplasia and squamous metaplasia. Stages (*a*) and (*b*) may therefore be analagous to the changes in the human tissue.

In addition to these changes in the bronchi of lung implants a previously unrecorded benign leiomyoma was found on the re-examination of an implant exposed

* Present address : Institute of Obstetric & Gynaecological Research, Royal Maternity Hospital, Rottenrow, Glasgow.

to 3,4 : 9,10-dibenzopyrene. This was in the form of a solid nodule at the centre of the lung, from which it appeared to originate (Fig. 5).

The high incidence of squamous tumours might have been due to the type of carcinogen used. To test this possibility, further implants were made using the following two unrelated substances each of which has been reported as producing experimental lung tumours in adult mice.

*Isoniazid*

There are several reports that a high incidence of lung tumours follows the administration of isoniazid to adult mice (Juhasz, Balo and Kendrey, 1957 ; Mori, Yasuno and Matsumoto, 1960 ; Biancifiori, 1961). As this is a well established drug in the treatment of tuberculosis these reports are of some significance.

*Urethane*

The intraperitoneal injection of mice with urethane gives rise to a high incidence of pulmonary tumours (Nettleship and Henshaw, 1943). If mice are injected when pregnant then the offspring also have a high lung tumour incidence (Smith and Rous, 1948). This shows that urethane crosses the placental barrier to affect the foetus in utero.

EXPERIMENTAL PROCEDURE

It was decided to inject solutions of both these substances into separate groups of pregnant mice and subsequently make implants with the embryo lung tissue which had thus been exposed to their action.

Isoniazid was used as 1% aqueous solution. The maximum dose which could be given without causing foetal death in utero was 0·2 ml. If 0·5 ml. was given abortion usually occurred within a few hours.

Urethane was also used as a 1% aqueous solution. This was a deliberate reduction in the dose used by Smith and Rous (1948) in order not to produce anaesthesia in the animals.

For both substances the same injection schedules were used and all the animals killed within 48 hours of the first dose (Tables I and II). All mice were of the BALB/c strain and pregnant for the first time. Injections were made intraperitoneally when they were within 3 days of term. The implantation of embryo lung and the subsequent preparation of specimens after 16 weeks were as previously described elsewhere (Peacock and Dick, 1963).

*Histology of isoniazid series*

It will be seen from Table I that adenomas occurred in all three groups. In general these were solid spherical tumours similar to those seen in previous

TABLE I.—*Embryo Lung Implants Exposed to Isoniazid in utero*

| Injection times | Implants made | Implants recovered | Lesions present |
|---|---|---|---|
| 48 hours and 24 hours | 28 | 26 | 1+4a |
| 24 hours | 28 | 25 | 4+1a |
| 24 hours and 6 hours | 22 | 22 | 3+3a |

(a = adenoma)

TABLE II.—*Embryo Lung Implants Exposed to Urethane in Utero*

| Injection times | Implants made | Implants recovered | Lesions present |
|---|---|---|---|
| 48 hours and 24 hours | 14 | 11 | 5 |
| 24 hours | 8 | 7 | nil |
| 24 hours and 6 hours | 14 | 13 | 8 |

experiments. There was one exception in which the tumour grew as a polyp into the lumen of a bronchus (Fig. 6).

Epithelial pearls of the type described earlier were present in two of the implants. Associated with these was an appearance not previously noticed, marked dilatation of the smaller bronchi and their surrounding alveoli, due to the stimulation of secretion on the part of the unaltered bronchial mucosa (Fig. 7). This appearance was also seen in other specimens from this group.

The most interesting finding was that in six cases there was a benign leiomyoma present of the type described in the preceding part of this paper. In these tumours there was direct continuity with the foetal lung with no evidence of origin from host tissues (Fig. 8). Special preparations confirmed the smooth muscle origin of these tumours. It is considered that they are most likely to have arisen from the smooth muscle surrounding the bronchi.

*Histology of urethane series*

In contrast to the previous group no lesions of any kind were found in those implants which had only been exposed to a single maternal injection. In the other two groups the findings were as follows.

In four specimens further leiomyomas occurred. These were in every way identical to the seven already recorded.

The presence of dense foci of lymphocytic infiltration in the lung was common,

EXPLANATION OF PLATES

FIG. 1.—Lung implant showing a small bronchus and its branches in which widespread changes in the epithelium are seen. The mucus secreting columnar epithelium is replaced by several layers of cuboidal cells. This implant and those in Fig. 2, 3, 4, were all exposed to 3,4 : 9,10-dibenzopyrene. H. & E. ×180.

FIG. 2.—A cross section of a bronchus in a lung implant showing the focal type of epithelial change affecting part of the circumference only. This type of appearance is commoner than that in Fig. 1. H. & E. ×180.

FIG. 3.—A small but well formed squamous pearl almost filling the bronchial lumen. H. & E. ×470.

FIG. 4.—A squamous pearl similar to that in Fig. 3 which has undergone hyalinization and completely occludes the bronchus. H. & E. ×180.

FIG. 5.—Part of the same implant as in Fig. 1 showing the compression of lung tissue by a benign leiomyoma arising from the embryo tissue. H. & E. ×180.

FIG. 6.—Lung implant exposed to isoniazid showing an adenoma growing into the lumen of a small bronchus to form a polyp like structure. H. & E. ×180.

FIG. 7.—Part of a lung implant exposed to isoniazid showing gross dilatation of bronchi due to stimulation of secretion. H. & E. ×180.

FIG. 8.—Part of a benign leiomyoma growing in close apposition to the host leg muscle. The striations of the skeletal muscle are clearly seen and help in differentiating it from the embryo tissue. H. & E. ×570.

1 2 3 4

Peacock.

5

6

7

8

Peacock.

either as a diffuse sheet of cells, or as focal concentrations. This was a type of reaction confined to this experiment and presumably a reflection of the known effect of urethane on the reticulo-endothelial tissue of the foetus.

In two specimens some thymic tissue had accidentally been implanted with the lung. This had hypertrophied to become 6 to 8 times the size of a control, but was otherwise similar in appearance.

Rather surprisingly not a single adenoma arose in this series, contrary to what had been expected. The greatly reduced dose level, and the shorter time for development must account for this difference from the results of Smith and Rous (1948).

### CONCLUSIONS

The recognition of a leiomyoma in a total of eleven lung implants makes altogether four distinct tumour types recorded in this tissue under these experimental conditions.

In the isoniazid experiments evidence of changes of the type held to precede the formation of squamous carcinoma were seen, namely the formation of epithelial pearls.

Confirmation was obtained in the urethane experiments of the ability of this substance to cross the placental barrier and affect the foetus, as shown by the lymphocytic infiltration of the implants. The dose used was one tenth of that of Rous and Smith's experiments.

Considered together these results suggest that embryo lung is so far the most sensitive of the foetal tissues used in this procedure for detecting early carcinogenic or toxic effects.

### SUMMARY

The development of squamous carcinoma in lung embryo implants according to a previously described technique is shown to be preceded by squamous metaplasia in the bronchial mucosa, and the formation of epithelial pearls within the lumen.

Exposures of similar implants to isoniazid and urethane by a technique of maternal injection gave rise to a number of benign leiomyomas. This makes a total of four types of tumour recorded in lung implants.

The presence of other non-neoplastic changes in the tissues of these implants further demonstrates the sensitivity of embryo lung in this test technique.

It is to be emphasized that all the results were obtained with very much smaller amounts of test material than in most toxicity tests.

This work was in part carried out while in receipt of a grant from the British Empire Cancer Campaign for Research.

### REFERENCES

AUERBACH, O.—(1956) *Cancer*, **9**, 76.
BIANCIFIORI, C.—(1961) *Proc. int. Conf. on Morphological Precursors of Cancer*, Perugia.
JUHASZ, J., BALO, J. AND KENDREY, G.—(1957) *Z. Krebsforsch.*. **62**, 76.
MORI, K., YASUNO, A. AND MATSUMOTO, K.—(1960) *Gann*, **51**, 83.
NETTLESHIP, A. AND HENSHAW, P. S.—(1943) *J. natn. Cancer Inst.*, **4**, 309.
PEACOCK, P. M.—(1962) *Br. J. Cancer*, **16**, 701
PEACOCK, P. M. AND DICK, E.—(1963) *Ibid.*, **17**, 59.
SHIMKIN, M. B.—(1955) *Adv. Cancer Res.*, **3**, 223.
SMITH, W. E. AND ROUS. P.—(1948) *J. exp. Med.*, **88**, 529.